# Cardiovascular magnetic resonance at 3.0T: Current state of the art

**DOI:** 10.1186/1532-429X-12-55

**Published:** 2010-10-07

**Authors:** John N Oshinski, Jana G Delfino, Puneet Sharma, Ahmed M Gharib, Roderic I Pettigrew

**Affiliations:** 1 Department of Radiology, Emory University School of Medicine, 1364 Clifton Road, Room AG34, Atlanta, GA 30322, USA; 2 Department of Biomedical Engineering, Emory University and the Georgia Institute of Technology, 101 Woodruff Circle Woodruff Memorial Building, Suite 2001, Atlanta, Georgia 30322, USA; 3 Laboratory of Integrative Cardiovascular Imaging, Department of Radiology, National Institute of Diabetes and Digestive and Kidney Diseases, National Institutes of Health, Clinical Research Center, Bldg. 10, Rm. 3-5340, MSC 1263, 10 Center Dr., Bethesda, MD 20892, USA

## Abstract

There are advantages to conducting cardiovascular magnetic resonance (CMR) studies at a field strength of 3.0 Telsa, including the increase in bulk magnetization, the increase in frequency separation of off-resonance spins, and the increase in T1 of many tissues. However, there are significant challenges to routinely performing CMR at 3.0T, including the reduction in main magnetic field homogeneity, the increase in RF power deposition, and the increase in susceptibility-based artifacts.

In this review, we outline the underlying physical effects that occur when imaging at higher fields, examine the practical results these effects have on the CMR applications, and examine methods used to compensate for these effects. Specifically, we will review cine imaging, MR coronary angiography, myocardial perfusion imaging, late gadolinium enhancement, and vascular wall imaging.

## Introduction

Three Tesla (3.0T) magnetic resonance imaging (MRI) scanners have been approved for use by the United States Food and Drug Administration (US FDA) since 1999 for head imaging and since 2001 for whole body imaging. 3.0T systems have become the standard for neurological imaging at many institutions [1-3]. The adoption of 3.0T for body applications, and specifically for cardiac applications, has been somewhat slower. The slower acceptance of 3.0T for cardiac applications is due to the unique challenges posed by cardiac imaging: the requirement of a large field of view, the motion of the heart, the position of the heart within the body, the proximity of the heart to the lungs, and high radiofrequency (RF) power deposition required in many high speed cardiac imaging sequences [4-8].

There are several advantages which motivate users to perform cardiovascular magnetic resonance (CMR) at a higher magnetic field strength. First, the bulk magnetization increases as the magnetic field strength is increased. The increased magnetization results in a theoretical increase in the signal-to-noise ratio (SNR) that is linearly related to field strength [9-11]. Second, increasing the magnetic field strength increases frequency separation of off-resonance spins. Therefore, the frequency difference between various hydrogen-based compounds is increased. The enhanced frequency differences may be exploited for improvement in spectroscopic imaging and potentially in fat suppression. A third difference that may be seen as an advantage is that increasing the main magnetic field increases the T1 of many tissues, with a negligible effect on the T2 [12-16]. The increase in T1 can have beneficial effects in some applications such as myocardial tagging and myocardial perfusion sequences, but requires attention to timing parameters in other sequences, such as late gadolinium enhancement.

There are challenges to routinely performing CMR at 3.0T. The homogeneity of the main magnetic field (B_0_) becomes more critical at 3.0T as off-resonance effects become important in many imaging sequences. The RF power deposition required for a given flip angle goes up with the square of the main magnetic field strength, so RF power deposition becomes an important consideration in imaging [11,17]. Additionally, maintaining the homogeneity of the field generated by the RF pulse (B_1_) is more of a challenge at 3.0T. Finally, signal loss from susceptibility-based artifacts becomes more prominent at 3.0T. From a practical standpoint, many implants that have been tested and deemed "MR compatible" at 1.5T have not been examined at 3.0T.

The purpose of this paper is to: 1) outline the physical effects that occur when increasing the main magnetic field from 1.5T to 3.0T, 2) outline the practical results these physical effects have on the major applications of CMR, and 3) review the technologic advances used to overcome these physical effects. Specific CMR applications to be discussed include: cine imaging, MR coronary angiography, myocardial perfusion imaging, late gadolinium enhancement, and vascular wall imaging.

## Background

### Physical Effects of Increased Field Strength

For the purpose of this review, we will concentrate on five major practical consequences of imaging at higher field strengths: (1) the increase in the signal-to-noise ratio, (2) the increase in the Larmor frequency, (3) the changes in relaxation times, (4) the changes in RF power deposition and specific absorption rate (SAR), and (5) the greater impact of field inhomogeneity and susceptibility.

#### Magnetization and signal-to-noise ratio (SNR)

Certainly, one of the greatest motivations for performing CMR at 3.0T is the increased signal-to-noise ratio (SNR) due to the increased bulk magnetization. Since many cardiac applications have relatively low SNR, the increase in SNR could enhance the clinical utility of many CMR applications and potentially enable new applications. The theoretical gain in SNR going from imaging at 1.5T to 3.0T is a factor of two. In many neuro-imaging applications, this increase has been realized [17]. In cardiac and body applications, the reported increases in SNR have been application and sequence dependant. Often, changes in sequence parameters are required to adapt 1.5T sequences for 3.0T, and these changes mitigate the expected theoretical increase in SNR. This is especially true for sequences that require multiple rapid sequential RF pulses, such as steady-state free procession (SSFP) sequences. Often, the specific absorption rate (SAR) limits are exceeded at 3.0T using the parameter settings employed at 1.5T. To meet the SAR requirements, modifications of the sequence, such as a decrease in flip angle (which reduces SNR) or an increase in repetition time (which increases susceptibility effects) are required. Reducing the flip angle causes a "parameter-induced" reduction in SNR. Other effects, such as RF field inhomogeneity, increased susceptibility, and changes in T1 at 3.0T cause "physical-induced" reductions in SNR. These effects will be discussed in detail in the following sections.

One potential use of the increased SNR seen at 3.0T is to employ parallel imaging at 3.0T. Parallel imaging reduces imaging time by using the additional spatial encoding information provided by multiple receiver coils to reduce the number of encoding lines needed. However, the reduced imaging time decreases the SNR by a factor approximately equal to the square root of the reduction in acquisition time. For example, a reduction in scan time of 2 by parallel imaging would cause SNR to be reduced by at least a factor of or 1.4 times [18]. The increase in SNR gained at 3.0T can be used to offset this reduction in SNR seen with parallel imaging techniques. The pairing of 3.0T and parallel imaging could reduce scan time by a factor of two with a preservation of SNR at 1.5T values. The higher resonant frequencies at 3.0T have another potential advantage for parallel imaging. The higher frequencies allow a greater separation of the coil elements in the frequency domain. Other possible uses of the SNR increase seen at 3.0T would be to improve spatial resolution while keeping SNR values near their 1.5T level, or to increase SNR above 1.5T levels while keeping total scan time constant.

#### Larmor and RF Frequency changes

The resonant (or Larmor) frequency changes linearly with field strength. Therefore, the doubling of the magnetic field strength from 1.5T to 3.0T causes a doubling of the resonant frequency. The increase of the resonant frequency also increases the separation between the frequencies of individual hydrogen-based compounds. In clinical cardiac imaging, this increased separation will lead to a doubling of the frequency difference between the primary water frequency and the fat frequency. The increased difference in the fat and water frequency can cause alterations in the appearance of the so-called "India-ink" artifact at 3.0T. The India ink artifact is caused by very low signal in pixels which cross boundaries of tissues containing a combination of fat and water. These pixels experience a signal phase cancellation at certain echo times that make borders around these tissues appear dark as though they were outlined in India ink [8,19]. This effect is a function of the repetition time (TR) and echo time (TE) used in a particular sequence, and therefore, it may appear in sequences at 3.0T when it was not seen at 1.5T.

The increased separation between fat and water may also allow for improvement in chemical-shift based fat suppression at 3.0T, as the frequency of the fat suppression pulse is farther from the primary water frequency and therefore the fat suppression pulse is less likely to inadvertently partially suppress the water signal. Chemical shift artifacts, where the spatial positions of pixels containing fat are shifted relative to water containing pixels, will be more prominent at 3.0T, however, for most cardiac sequences, the effect is negligible. The increased frequency separation between hydrogen-based compounds has significant benefits for proton spectroscopy. The increased frequency separation should create less overlap in peaks in the spectra and therefore enable better quantification of specific compound concentrations. The increase in SNR will translate to higher spectral peaks relative to the background signal [14,15,20].

**Figure 1:**
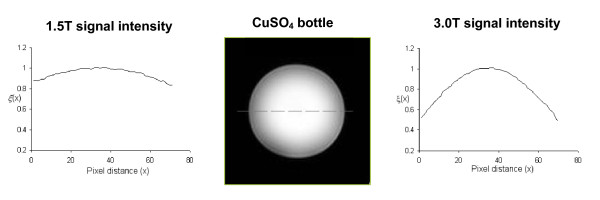
**The *field-focusing *effect is illustrated in a phantom image**. Relative signal intensity profiles (ξ) are shown as a function of position (x) in a CuSO_4 _bottle. On the left is a signal intensity profile for a 90° flip angle in a 1.5T scanner. Signal intensity is fairly uniform across the bottle. On the right is a signal intensity profile for a 90° flip angle in a 3.0T scanner. Signal intensity is *focused *at the center of the bottle.

The higher resonant frequency at 3.0T requires that the frequency of the RF excitation pulse change to match the 3.0T resonant frequency. At these higher resonant frequencies, significant spatial variations in the flip angle can be seen at 3.0T that are not seen at 1.5T. At main magnetic fields strengths of 3.0T and above, the electrical conductivity and permittivity of the body tissue as well as the shape of the body significantly affect the propagation of the magnetic fields. Additionally, the wavelength of the generated magnetic fields is on the order of the body's size. Together these effects cause the strength of RF field to vary with spatial position. The effect has been referred to as "field-focusing", because flip angles in head images are increased or "focused" near the center of the field of view. However, the effects can be quite variable and are not easily predicted, especially in cardiac imaging [9,21]. These effects can be ignored at 1.5T, but must be considered at 3.0T and above. Using standard pulses, the resultant flip angle across the body has been reported to vary by 40% and the variations are dependant on tissue type [21-24]. Figure 1 shows a CuSO_4 _bottle phantom at 1.5T and 3.0T after a 90° rf pulse. Much more spatial variation of the flip angle is seen at 3.0T.

#### Changes in RF Power and SAR

As a direct result of increasing the main magnetic field strength, the frequency of the RF pulse increases. The increase in frequency increases the power required to create a given flip angle by the square of the change in main magnetic field [9,11]. The specific absorption rate (SAR) is an estimate of the time-averaged power (either per gram of tissue or over the whole body), therefore SAR of a sequence at 3.0T will have four times the SAR of the identical sequence run at 1.5T. The SAR limits are set to prevent excessive heating of tissue due to RF power deposition. SAR cannot be directly measured in patients, so scanner manufacturers use computational models to estimate SAR for specific sequences. These models may vary between scanners, but 3.0T sequences will always have significantly higher SAR compared to sequences run at 1.5T. Practically, this will require sequences that use high levels of RF power deposition, such as SSFP, to be modified when implementing them at 3.0T to avoid exceeding SAR limits. The most common way to modify a sequence to reduce SAR is to reduce the flip angle, and reduction of flip angle will directly affect SNR. The use of variable-rate selective excitation (VERSE) or other tailored RF pulses can reduce SAR without directly reducing the flip angle [25-28]. Increasing TR will also reduce SAR, but increasing the TR will cause a greater increase in susceptibility artifacts, as will be discussed below.

#### Changes in Relaxation Times

Moving from 1.5T to 3.0T changes the T1 relaxation time in tissues. T1 relaxation times at 3.0T are generally longer than at 1.5T. Since the vast majority of CMR sequences have significant T1-weighting, the change in T1 values will affect image contrast and sequence parameters. The T1 of normal myocardium has been shown to increase between 12% to 42% going from 1.5T to 3.0T, and the T1 of blood has been shown to increase 7% to 40% when moving from 1.5T to 3.0T [12,29]. The variation in reported values is probably due to differences in physiology between individuals and differences in pulse sequences and methodologies used to measure T1.

In the range of 1.5T to 3.0T, the field dependence of T1 relaxation times for Gd-chelates is quite small [30,31]. Therefore, the T1 differences between 1.5T and 3.0T for blood that exist in the pre-contrast state are negligible early after contrast infusion [12]. The combination of longer T1's in most tissues at 3.0T and the relatively small effects of field strength on Gd-chelate's relaxivity create an advantage for T1-weighted contrast enhanced magnetic resonance angiography (MRA). The longer T1 in static tissue at 3.0T allows for greater background tissue suppression at 3.0T compared to 1.5T. The T1 reduction in the blood with high levels of Gd-chelates at 3.0T is preserved at 1.5T. This net effect of preserved shortening of blood T1 and improved background suppression is independent of SNR increases; this is one of the factors responsible for the excellent results seen for MRA at 3.0T [32].

Changes in T2 values at 3.0T are negligible for most tissues allowing minimal protocol changes for morphologic T2 spin-echo imaging [29]. However, T2* changes become increasingly significant at 3.0T for commonly used gradient-echo cardiac sequences. The reduction in T2* going from 1.5T to 3.0T is a factor of two, suggesting a linear dependence of T2* on field strength. More susceptibility artifacts have been reported with T2*-weighted imaging at 3.0T [33]. The reduction of T2* can be exploited at 3.0T in several ways. First, the blood oxygenation level dependant (BOLD) contrast is based on T2* effects and several studies have shown that the BOLD effect is increased at 3.0T compared to 1.5T [34,35]. Secondly, imaging of iron-based contrast agents has been shown to be more sensitive at 3.0T compared to 1.5T due to the role of iron on T2* relaxation, (greater iron concentration causes greater regional signal dephasing and signal loss) [34]. Third, T2* sensitivity to iron at 3.0T may be exploited to better delineate individuals with iron-overload as seen in patients with Thalassemia [36,37]. However, the quantification of actual T2* values in areas of very high iron concentration may be more difficult at 3.0T due to the very rapid decay of the signal [33].

#### Field Homogeneity and Susceptibility

**Figure 2:**
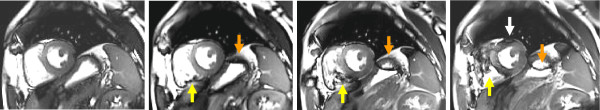
**Short-axis, SSFP images acquired at four different repetition times (TR's), ranging from 2.3msec (far left) to 5.0msec (far right)**. As TR is increased, artifacts due to susceptibility and field inhomogeneity are seen in the RV (yellow arrow), at the diaphragm (orange arrow), is and of the anterior and lateral walls of myocardium (white arrows).

The differences in magnetic susceptibilities of adjacent tissues cause local alterations in the main magnetic field. The magnetic field alterations due to susceptibility differences are a complex function of the local geometry, but are linearly related to the main magnetic field strength [11]. Increasing the magnetic field strength from 1.5T to 3.0T causes a linear increase in the susceptibility induced field variations [38,39]. These field perturbations will be most prominent at locations where there is a large difference in magnetic susceptibility between tissues, such as the myocardial-lung interface, or areas near the coronary sinus that contain de-oxygenated blood [39,40]. The variations in the main magnetic field due to susceptibility differences cause a change in the phase of the MR signal. These local phase changes due to susceptibility differences manifest themselves as signal loss in the image. In a gradient-echo sequence, the phase shift is linearly proportional to the susceptibility difference as well as the echo time (TE). In SSFP sequences, the phase shift is linearly proportional to the susceptibility difference and the repetition time (TR). Since TR > TE, the susceptibility induced signal loss artifacts are more prevalent in SSFP sequences, Figure 2.

The appearance and location of banding artifacts in SSFP images is also affected by field strength and susceptibility differences. The banding artifacts occur at specific frequencies where there is a positive to negative phase transition of the signal. In areas where susceptibility differences cause variations in the main magnetic field, banding artifacts can appear. At 3.0T, the susceptibility effect is greater and hence banding artifacts will be more prevalent. Since the susceptibility differences are proportional to field strength, SSFP sequences at 3.0T in areas where tissue susceptibility differences are large are particularly prone to artifacts. For CMR, a prominent location for signal loss and banding artifacts is the infero-lateral wall of the myocardium at the lung/diaphragm interface.

## Results

### CMR Applications at High Field Strengths

#### Cine Imaging of Cardiac Function

The use of balanced steady-state free precession (SSFP) imaging has become the cornerstone for cardiac functional analysis with MRI at 1.5T. SSFP provides significant improvements in SNR and CNR over standard segmented cine gradient-echo methods. At 1.5T, SSFP is less sensitive to inflow and turbulence-related flow voids than standard gradient-echo sequences. While implementation of SSFP at 3.0T is feasible with promising results shown by several groups [41-46], it remains an ongoing challenge to overcome the sensitivity of SSFP to high field-related artifacts.

#### SNR and CNR

Reproducible image quality for cine SSFP is important for robust evaluation of ventricular function, and with modifications to a 1.5T SSFP sequence, high quality functional cine images can be achieved at 3.0T. In comparison to 1.5T, cine SSFP has shown increased SNR for myocardium and blood at 3.0T, as well as increased blood-to-myocardium CNR. Increases in CNR have varied widely from 9.4% to 86% [41,42]. The differences in reported values are due to differences in acquisition parameters, the extent of shimming, coil positioning, the patient groups studied, and placement of ROI for signal measurements. Due to the potential greater variation in flip angles across the image and the greater effect of susceptibility at 3.0T, it is important to describe the variability of myocardium SNR segmentally, and between slices.

Comparative analyses of LV function indexes, mass, and volume using cine SSFP has shown no significant difference in values obtained at 3.0T compared to 1.5T [47,48]. Therefore, one can use existing knowledge and experience of MR quantification algorithms for 3.0T imaging. Compared to 1.5T, CNR between blood and myocardium is reduced in the RV at 3.0T relative to LV, which may affect reproducibility of RV functional evaluation [47].

#### Frequency, Relaxation Time, Homogeneity, and Susceptibility

One of the most commonly seen artifact in 3.0T SSFP imaging is a "dark-band" artifact described earlier. Dark band locations are related to the local field inhomogeneities and the sequence TR. For similar TR's, heightened local field inhomogeneities at 3.0T may cause the dark bands to come into close proximity of the imaging region-of-interest, potentially resulting in severe image deterioration. Reducing TR widens the band spacing, alleviating the presence of dark bands in the image, insomuch that concurrent increases in bandwidth and SAR are tolerable. A fast, frequency-scout acquisition can be utilized to determine the optimal resonance frequency offset to incorporate with SSFP imaging [49,50]. This "frequency scout" offers a visual indication of the resonance offset to be employed, but it requires an additional acquisition, which adds to exam time. A frequency offset based on the frequency scout image, usually on the order of +/- 200Hz, shifts dark band artifacts away from the imaging region-of-interest. Alternatively, overcoming local field inhomogeneity with a higher-order shim routine offers the clearest benefits to high quality cine SSFP imaging at 3.0T [44]. Use of a shim routine based on a field map of the heart combined with a frequency scout acquisition has shown excellent results. Other methods to address banding artifacts on cine SSFP at 3.0T are being investigated, such as wideband SSFP, which uses sequence adaptation to widen the spacing between dark bands without resonance frequency modification [50].

The use of fast, segmented, spoiled gradient echo (GRE) imaging is prevalent in cardiac imaging, with applications including late gadolinium enhancement imaging, perfusion imaging and MR angiography. After the development of SSFP, use of GRE for functional evaluation has been limited to specific applications, such as quantitative flow imaging and regional detection of flow turbulence and regurgitation. Since the SNR of cine GRE imaging relies predominantly on the steady-state T1 signal of blood and myocardium, there will presumably be reduced SNR at 3.0T for identical pulse sequences due to lengthening of T1 relaxation times. Although this has been revealed experimentally [41], the resulting contrast-to-noise ratio and overall image quality of GRE has been shown to improve at 3.0T compared to 1.5T when using optimized sequences [45]. Cine GRE imaging benefits from its relative insensitivity to field inhomogeneity artifacts in comparison to SSFP. This makes imaging of cardiac function with GRE a reliable option at 3.0T. However, unbalanced gradient rephasing and inflow phenomena may lead to transient signal and flow voids within the ventricle [51]. The use of standard GRE sequences at 3.0T has been examined in the presence of an extra cellular gadolinium-based contrast agent. Hamdan et al showed that long-axis image quality improved after contrast infusion, but short axis images did not improve. The improvement in image quality in the long axis images was due to a reduction in in-plane flow dephasing artifacts. Differences in LV volumes and EF's were also seen between pre-and post-contrast scans. The differences were due to differences in the conspicuity of trabeculae and papillary muscles before and after contrast [51].

#### Myocardial Tagging

**Figure 3:**
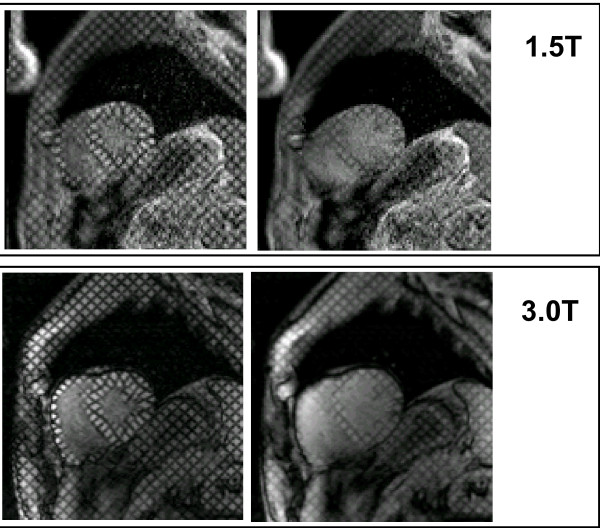
**Tagging images acquired in systole and mid-diastole at 3.0T and 1.5T**. Note that in diastole tag lines have faded more at 1.5T than at 3.0T. The reduced fading at 3.0T is due to the prolonged T1 at 3.0T. The longer lasting tag lines may allow for better analysis of diastolic function at 3.0T. Signal-to-noise is also higher in the 3.0T images.

Myocardial "tagging" is a method in which RF pulses and gradients are used to pre-saturate magnetization perpendicular to the slice plane prior to cine imaging. This pre-excitation pulse suppresses magnetization locally to create "tag lines", so that regional contractile motion can be visualized over the cardiac cycle. Tag lines can be created in a radial, line, or grid pattern. This technique allows the quantification of myocardium strain using computer-assisted programs to track the displacement of tag intersection points over the cardiac cycle. Since the saturated magnetization in the tag line is subject to T1 relaxation over the course of the cardiac cycle, the tag lines gradually fade by diastole. This tag fading is a common feature at 1.5T, since the T1 of myocardium is approximately 800-900ms, which is on the order of one cardiac cycle. At 3.0T, this tag fading is lessened since T1 is longer, allowing tag lines to persist over more of the cardiac cycle. Initial reports have shown improvement of tag line persistence in the LV by 37% [42]. The tag line persistence is complemented by improved SNR and CNR of the cine gradient echo acquisition. These qualitative improvements at 3.0T translate directly to improved quantitative analysis of myocardial tagging, allowing better detection of tag lines and better depiction of epicardial and endocardial borders for processing multi-directional strain, Figure 3. Implementation of myocardial tagging has also been done using SSFP imaging for signal readout, but application at 3.0T has been limited [52-54].

It is now clear from multiple studies that significant increases in SNR and CNR are gained when performing cine imaging at 3.0T. However, when using SSFP imaging at 3.0T, the presence of artifacts due to susceptibility and banding are more prevalent compared to 1.5T. Strategies to quickly and easily reduce the artifacts are needed for 3.0T SSFP imaging to completely replace 1.5T imaging on a widespread basis.

#### MR Coronary Angiography

MR coronary angiography is challenging at any field strength, as the small vessel size and motion of the heart necessitate rapid imaging and high spatial resolution. At 1.5T, coronary angiography suffers from SNR limitations. Imaging at 3.0T could potentially mitigate these SNR concerns. The increased SNR at 3.0T combined with parallel imaging have caused renewed interest in using a 3 D volume that covers the whole heart in a single scan, making it possible to visualize the entire coronary tree [55-57]. Voxel sizes 50% smaller than those employed at 1.5T have been implemented at 3.0T with preserved image quality [58]. Alternately, voxel sizes can be kept at 1.5T sizes and the increased SNR available at 3.0T can be used solely to reduce image acquisition time with parallel imaging [59].

#### SNR and CNR

Several studies have shown that imaging of the coronary arteries is feasible at 3.0T. These studies have been conducted using a variety of different pulse sequences including segmented gradient echo (GRE) sequences [55,57,58,60,61], fast spin echo sequences [62], and recently, SSFP sequences [63,64]. There have been two studies directly comparing non-contrast coronary angiography images acquired at 1.5 and 3.0T. Both these studies have demonstrated improved SNR and CNR after moving to 3.0T. Yang et al reported an average SNR increase of 47% and a CNR increase of 36% when moving from 1.5 to 3.0T using a high-resolution interleaved spiral GRE sequence [65]. Bi et al reported SNR and CNR increases of 87% and 83% respectively when moving from 1.5T to 3.0T using a high-resolution three-dimensional multislab SSFP sequence, although greater image artifacts were also observed at 3.0T [66].

**Figure 4:**
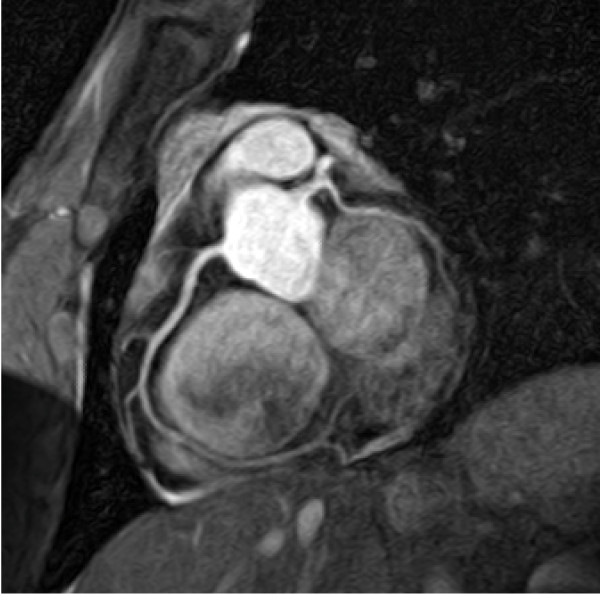
**Image of the coronary arteries obtained at 3.0T using a 32-channel coil and employing a targeted 3 D GRE approach**. Pixel resolution is 0.7 × 1.0 × 2.0. The right coronary artery (RCA) and left circumflex artery (LCX) and branching vessels can easily be seen. In general, the 3.0T coronary images have superior SNR and CNR compared to 1.5T.

A few reports have examined contrast-enhanced coronary angiography at 3.0T although a direct comparison with 1.5T has not been done. In a study of 9 healthy volunteers, Bi et al used a three-dimensional, inversion recovery prepared GRE sequence during contrast infusion [55]. A 53% increase in SNR and a 305% increase in CNR was observed compared to a comparable non-IR prepared sequence acquired without contrast [55]. In addition, the mean measured length of both the LAD and the RCA was significantly greater in the contrast-enhanced images. Kotys et al described a bilateral shadowing artifact along the margins of the coronary arteries when using a high relaxivity contrast agent at 3.0T. Delaying acquisition until the contrast agent has reached steady-state and imaging with the more time efficient centric radial order gave optimal contrast enhancement, but lead to overestimation of the vessel width [67], Figure 4.

#### Frequency, Relaxation Time, Homogeneity, and Susceptibility

The greater spectral separation of fat and water may enable more efficient fat-suppression pulses at 3.0T which may improve coronary imaging. Since signal from fatty tissue surrounding the coronaries is often suppressed using a frequency-selective, fat-suppression pulse [68], the greater spectral separation between fat and water at 3.0T may improve visualization of the coronary arteries by more efficiently suppressing the fat signal around the vessels. Alternately, the better spectral selectivity can be used to shorten the duration of the spectrally-selective contrast preparation pulse. This will reduce sequence TE and minimize susceptibility artifacts and T_2_* signal loss in the images [59,65].

RF field inhomogeneity at 3.0T can cause local enhancements in signal intensity in different regions. This local brightening has been observed in coronary MRA scans, depending on the coils used [66]. One possible solution for reducing such signal variation is to use adiabatic RF pulses that are designed to reduce B1 inhomogeneities [66].

Main magnetic field inhomogeneity is more pronounced at 3.0T than 1.5T especially when imaging over a large volume such as is done in whole heart coronary MRA. Volumetric shimming over the heart can mitigate inhomogeneity effects at 3.0T [68]. To generate greater magnetic field uniformity, a higher order volumetric shim calibration, followed by a dynamic, real-time, multi-slice linear shim to individually fine-tune the shim for each slice before image acquisition can be used [59]. Greater than 40% reductions in magnetic field inhomogeneities have been demonstrated when higher order shimming was applied (average RMS field deviation decreased from 61.2+/-3.2 Hz without shimming to 16.5+/-1.3Hz when higher-order terms were used) [69].

Susceptibility artifacts increase at 3.0T due to shorter T2* and greater field inhomogeneities, and these artifacts may obscure coronary vessels along the heart-lung interface [65]. In a direct comparison of MR coronary angiography at 1.5 and 3.0T, Yang et al noted susceptibility artifacts were present in 9/23 subjects at 3.0T, but in none of the subjects at 1.5T [65]. Use of the shortest TE and readout gradients possible can help reduce the effects of susceptibility artifacts [59,65]. Furthermore, careful shimming can further reduce susceptibility artifacts [56]. It is important to note that although susceptibility artifacts were more prevalent at 3.0T in the previously mentioned study, overall image quality for all the coronary segments was actually higher at 3.0T [65].

At 1.5T, three-dimensional SSFP imaging is the preferred method for the imaging of the coronary arteries [68]. However, SSFP techniques cannot be directly translated to 3.0T as the higher frequencies of the RF pulses mean that SAR limits are reached much more rapidly. To implement SSFP sequences at 3.0T, flip angles must be adjusted and/or repetition times increased. Obtaining consistent results when imaging the coronary arteries using SSFP sequences have proved difficult at 3.0T due to the high sensitivity of SSFP to off-resonance artifacts, and it has been suggested that better image quality may be obtained more consistently by using a spoiled GRE sequence instead of SSFP [60,61].

3.0T has caused renewed interest in MR coronary angiography and improvements in coronary imaging have been seen at 3.0T compared to 1.5T. The optimization of coronary imaging at 3.0T is continuing.

#### Myocardial Perfusion

Myocardial perfusion imaging is critical in determining the extent and location of regional ischemia. Recent studies have shown that myocardial perfusion imaging with MRI employing a gadolinium-based contrast agent is comparable to nuclear based techniques such as SPECT [64,70]. Imaging at 3.0T offers potential benefits for myocardial perfusion imaging over 1.5T.

#### SNR and CNR

Since myocardial perfusion imaging requires rapid acquisition, SNR and CNR are often compromised at 1.5T. Spatial resolution is often sacrificed for temporal resolution, which can lead to ringing artifacts in the image that can be misinterpreted as perfusion defects [56]. Moving from 1.5T to 3.0T theoretically produces a doubling of SNR, and therefore perfusion imaging at 3.0T may have practical advantages over imaging at 1.5T. The higher SNR at 3.0T can be used to increase either spatial or temporal resolution or can be applied to parallel imaging techniques that decrease image acquisition time [56]. Deciding which is the best choice will depend on the specific application. Better temporal resolution is critical in stress perfusion studies, so the added SNR may be best applied to increase the temporal resolution of stress perfusion studies [70]. Longitudinal relaxation time (T1) of myocardial tissue increases with higher field strength and the relaxivity of most gadolinium-based contrast agents does not change greatly with field strength [56]. This translates into potentially increased contrast between perfused and non-perfused myocardial tissue and higher CNR for perfusion images acquired at 3.0T [56]

Several studies have reported significant increases in SNR, CNR, and overall image quality for perfusion imaging when moving from 1.5 to 3.0T [42,71]. Gutberlet et al reported an SNR increase of 109% when moving from 1.5T to 3.0T with a T1-weighted segmented EPI sequence [42]. Aroz et al reported that SNR increased from 25+/-8 to 82+/-26 for peak myocardial enhancement during first-pass perfusion imaging when moving from 1.5T to 3.0T [71]. Plein et al showed that image quality is comparable to 1.5T when a five-fold kt-SENSE reduction factor is used at 3.0T [72]. Christian et al showed in an animal study that SNR and CNR were better in perfusion images acquired at 3.0T compared to those acquired at 1.5T. Correlation between actual flow assessed with microspheres and absolute blood flow assessed by MRI was better at 3.0T than 1.5T [73]. However, it is important to note that the SNR and CNR increases observed between 1.5 and 3.0T depend on the sequence and specific imaging parameters employed. Diagnostic accuracy of myocardial stress perfusion imaging for detecting hemodynamically relevant coronary artery stenosis at 3.0T has been reported as between 84-86%, which is comparable to previously reported values of 82-89% at 1.5T [74-76]. Diagnostic accuracy of perfusion imaging for detecting myocardial ischemia between 1.5T and 3.0T has not been directly compared.

#### Frequency, Relaxation Time, Homogeneity, and Susceptibility

Most centers performing perfusion imaging at 1.5T use a non-selective saturation recovery (SR) 90° RF pulse rather than an inversion recovery (IR) 180° RF pulse to generate contrast between the hypo-perfused and normally-perfused tissue. Although more contrast can theoretically be generated with an IR pulse, the increased speed, reduced heart rate dependence, and more consistent slice to slice contrast have made SR imaging the current standard. Use of SR-based protocols is even more crucial at 3.0T compared to 1.5T. The regional RF inhomogeniety due to field focusing at 3.0T will be less evident in a saturation pulse than an inversion pulse. Although the effect can also be seen in a saturation pulse, due to the lower flip angle, the effect will be less significant. Adiabatic B_1_-insensitive rotation pulses using phase cycling (BIR-4) or pulse trains can be used to increase the spatial homogeneity of the saturation pulse over the heart [56,77]. A recent study has reported successful acquisition of perfusion images at 3.0T using a 3 D gradient echo sequence preceded by a 90° global adiabatic saturation pulse [78].

The arguments for using a GRE readout versus an SSFP readout are similar to the arguments for using a saturation pre-pulse versus an inversion pre-pulse. Most studies at 1.5T are carried out with a GRE readout, although good results have also been obtained using a SSFP readout [79]. The greater susceptibility artifacts of the SSFP at 3.0T make GRE the usual choice for 3.0T perfusion imaging.

Perfusion imaging is playing an increasing role in evaluation of patients with suspected ischemia undergoing cardiac MR exams. Use of a saturation recovery sequence with standard gradient echo readout allows for the minimization of susceptibility artifacts at 3.0T and allows one to take advantage of potential SNR and CNR gains for increased magnetization as well as gains from the increase in T1 of myocardial tissue.

#### Late Gadolinium Enhancement

Late Gadolinium enhancement (LGE) imaging is done using an inversion-recovery (IR) prepared gradient echo sequence. The sequence depends on T1 recovery after the inversion pulse to generate contrast between infarcted myocardium containing gadolinium and normal tissue. Because the sequence generates image contrast based on T1, special attention needs to be paid to timing parameters when performing LGE at 3.0T. As with all sequences at 3.0T, there are SNR advantages to performing LGE at 3.0T.

#### SNR and CNR

Several studies have compared LGE at 1.5T and 3.0T. Klumpp, et al compared 20 subjects imaged at 3.0T and a separate set of 20 subjects imaged 1.5T [80]. All subjects had chronic MI and there were no differences between the numbers of segments with LGE in patients imaged at each field strength. A segmented, IR-prepared, GRE sequence was used for imaging, and the inversion time (TI) was optimized for each patient using a TI scout sequence [81]. Imaging parameters were otherwise held constant for the two field strengths, and SNR and CNR were compared. The study found that SNR in the infarcted region at 3.0T was increased 1.6 times compared to 1.5T, and CNR between normal and infarcted myocardium at 3.0T increased 1.9 times when compared to 1.5T. Huber, et al studied 10 subjects with chronic MI at both 1.5T and 3.0T [82]. A single shot, phase-sensitive inversion recovery (PSIR) sequence was compared at the two field strengths. A TI scout sequence was used to determine the inversion time for each patient. In the magnitude images, CNR between the infarcted and normal myocardium was 2.1 times higher at 3.0T compared to 1.5T. Infarct volume between 1.5T and 3.0T correlated well but there was some significant variation in individual patients. Bauner et al imaged 15 patients with chronic MI at 1.5T and 3.0T [83]. LGE imaging was done with a segmented, IR-prepared, GRE sequence and optimal TI time to null myocardium was determined using a TI scout sequence. SNR of the infarcted myocardium increased by 1.8 times at 3.0T compared to 1.5T. CNR between infarcted and normal myocardium increased by 2 times at 3.0T compared to 1.5T. Infarct volume compared well over all subjects, but Bland-Altman analysis showed some significant differences between individual patients. Ligabue et al studied 35 consecutive patients with acute MI at 1.5T and 3.0T [84]. LGE imaging was done by an IR-prepared, segmented, GRE sequence and the TI for nulling myocardium was found by a Look-Locker sequence. SNR in infarcted myocardium was increased 3.9 times at 3.0T compared to 1.5T. CNR was increased 3.3 times at 3.0T compared to 1.5T.

These studies all show that there appears to be a significant increase in SNR of infarcted myocardium, and an increase in CNR between normal and infarcted myocardium. This increase in SNR is specifically due to the increase in bulk magnetization going from 1.5T to 3.0T. The majority of studies have used a segmented gradient-echo technique for readout. Similar increases are seen using a balanced SSFP readout, but more image artifacts were reported when the SSFP readout was used [82].

#### Frequency, Relaxation Time, Homogeneity, and Susceptibility

The infusion of a gadolinium chelate based contrast agent changes the T1 of the blood and myocardium as a function of the concentration of the contrast agent in the tissue. For the clinical doses used, the relaxation time of the contrast agent dominates over the intrinsic relaxation time of the blood and perfused tissue. Due to the nearly field independent T1 relaxation values of gadolinium chelates in the 1.5T-3.0T range, soon after infusion there is no difference in relaxation times in the blood pool or myocardium at 1.5T and 3.0T [12]. However, as the contrast agent is cleared from the normal myocardium, the field dependent T1 difference in the myocardium is again seen. At 15-40 minutes post-contrast infusion, the T1 of normal myocardium which has cleared the majority of the contrast agent is again longer at 3.0T than 1.5T. This field dependent T1 contrast will cause the inversion times to null normal myocardium to be longer at 3.0T than at 1.5T. In a study of subjects with chronic MI, Klumpp et al found that TI times to null normal myocardium were 260 ± 30 sec at 1.5T and 330 ± 48 sec at 3.0T [80]. In a study of patients with acute MI, Ligabue et al found that TI was 330 ± 50 at 1.5T and 375 ± 55 at 3.0T [84]. These studies indicate there is a lengthening of the TI time required to null normal myocardium, but the exact difference between 1.5T and 3.0T will depend on the contrast agent dose and the time after infusion that imaging is conducted, as well as the individual physiology of specific patients. The prolonged T1 of normal myocardium at 3.0T also theoretically increases the available contrast between infarcted and normal myocardium. The increased CNR is partly due greater signal recovery in infarcted myocardium containing the gadolinium chelate based contrast agent when the inversion time for nulling normal myocardium is increased. The amount of this CNR increase will depend on the imaging sequence used, heart rate, and contrast dose, but this effect may contribute to the higher levels of CNR increase seen in LGE imaging at 3.0T.

The RF pulse inhomogeneity affects image quality of LGE at 3.0T more than 1.5T. The variation in the flip angle can cause the inversion pulse to vary across the myocardium and blood pool, potentially yielding a flip angle greater than 180° in some parts of the heart or blood pool and less than 180° in other locations. The result of inhomogeneous RF pulses is incomplete suppression of the myocardium, or spatially varying suppression of the myocardium and blood pool. Use of tailored RF pulses or adiabatic inversion pulses reduce this field focusing effect significantly and produce more spatially homogeneous inversion. As mentioned previously, the RF power required to produce an inversion pulse at 3.0T is four times greater than the power needed to create the inversion pulse at 1.5T. This can result in increased RF power deposition in the patient. However, with the long times between inversion pulses in LGE imaging (1-3 heart beats), the increased power seldom causes issues with SAR limitations.

In general, LGE at 3.0T has proven to be as effective as it has been at 1.5T. SNR as well as infarct-to-myocardium contrast are superior at 3.0T, and some inherent CNR advantages exist for imaging infarcts at 3.0T. The user must be aware that timing parameters for nulling myocardium will change at 3.0T.

#### Vessel wall imaging

**Figure 5:**
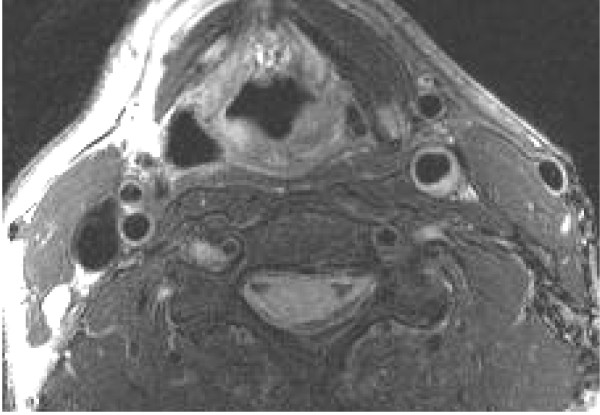
**Image of the carotid artery obtained in a subject with a thickened wall in the left common carotid artery**. The image was acquired at 3.0T with a four-channel carotid coil. Image quality as assessed by SNR and CNR is superior to 1.5T images

Intimal-medial thickness (IMT) has been used as a marker of risk for adverse cardiovascular events and the presence of specific plaque components such as necrotic core has been shown to be important in identifying vulnerable plaques. The reduction or progression of IMT or change in plaque components has been used as an endpoint for judging the efficiency of pharmacologic interventional therapies [85,86]. For studies designed to assess changes in vascular wall thickness in response to an intervention, image quality, SNR and CNR are important. Increased SNR, CNR, and image quality lead to better inter-test and inter-observer reproducibility, which allows interventions to be evaluated in a smaller number of subjects, or allows smaller effects to be observed, figure 5.

The increased SNR offered by 3.0T has been studied for its effect on increasing reproducibility of vessel wall area, vessel wall volume, and mean wall thickness measurements. In a study of six healthy volunteers, Kokzoglou et al found a 223% increase in SNR for the carotid wall at 3.0T compared with 1.5T. CNR between the wall and lumen increased 255%. Imaging was done with a multi-slice, black-blood, dual-inversion, turbo spin-echo (TSE) sequence. Both 1.5T and 3.0T scanners were the same manufacturer, but coil size and imaging parameter varied slightly between field strengths [87]. In a study of 10 healthy volunteers, Dehnavi et al examined the inter-test and inter-observer reproducibility of vessel wall area and total lumen area measurements. The relative error between repeated scans was 2.6% with interclass correlation of 0.98, a significant improvement over the relative errors seen at 1.5T [88]. Similar results were seen in a study by Syed et al that examined 10 subjects at 3.0T who had IMT >0.65 mm by ultrasound. Subjects were imaged two times using dual-inversion, black-blood, TSE and using a carotid coil. Inter-class correlation was 0.98 for total vessel volume [81]. A study by Yarnykh et al examined five healthy subjects and two patients at 1.5T and 3.0T. The 1.5T and 3.0T MR scanners were made by the same manufacturer and coils used were similar. Dual-inversion, black-blood TSE sequences with similar scan parameters were used at both field strengths. SNR increased 77 ± 44% for T2-weighted images, and CNR between the wall and lumen increased 82 ± 46%. No difference between vessel wall area measurements between 1.5T and 3.0T were noted, suggesting 1.5T and 3.0T can be used interchangeably in studies [89].

3.0T has also been examined for its ability to improve plaque component identification compared to 1.5T. Underhill et al compared the ability of 1.5T and 3.0T to identify carotid plaque components in 20 subjects with moderate carotid stenoses. Multiple contrast mechanisms were combined to identify plaque components. Importantly, two signal averages were used at 1.5T, and only a single signal average was used at 3.0T. Despite these differences in number of signal averages, 3.0T images showed an increase of SNR of ≈20% over 1.5T images. Morphometric variables such as total vessel wall area and lumen agreed well between 1.5T and 3.0T, having inter-class coefficients of 0.88 to 0.96. This study again suggests 1.5T and 3.0T can be used interchangeably for morphometric measurements [90].

A study by Vidal et al imaged eight subjects with IMT ≥ 0.5 mm by ultrasound. Each subject was scanned two times at each field strength, using T2-weighted, dual inversion, black-blood sequences. Both 1.5T and 3.0T scanners were made by the same manufacturer, and the coils used were virtually identical. The study found a 90% increase in SNR going from 1.5T to 3.0T and a 25% increase in CNR. Although SNR increased, the study found no improvement in reproducibility at 3.0T compared to 1.5T with the coefficient of variance being 7.8% and 5.7% respectively. In addition, this study found that there was a significant difference between vessel wall volumes measured at 1.5T compared with 3.0T. Averaged over all 8 subjects, measurements of vessel wall volume at 1.5T were approximately 10% higher at 1.5T. Data was not presented to assess if this difference was consistent over all subjects [91].

Increased SNR at 3.0T may improve vessel wall imaging in the aorta. In a study of 32 subjects (20 healthy volunteers and 12 subjects with cardiac disease), Maroules et al found that SNR increased ≈50% and CNR increased ≈70%. Differences in mean wall thickness measurements between 1.5T and 3.0T were not significant, even with the addition of parallel imaging at 3.0T. Roes et al evaluated navigator-echo gated 3 D, dual inversion recovery, black-blood imaging for determining aortic wall thickness [92]. Seven healthy subjects were imaged two times and SNR, CNR, and reproducibility of vessel wall volume were evaluated. The use of the respiratory navigator reduced breathing artifacts as evidenced by an increase in vessel sharpness for the navigator gated sequence over the non-gated sequence. The ICC correlation for inter-scan reproducibility was 0.95, and the CV was 5.8%.

The increased T1 values of blood at 3.0T compared to 1.5T will require a change in the inversion time (TI) in order to null blood. The required changes can be estimated from T1 measurement of blood at 3.0T.

The use of 3.0T for vessel wall imaging in the carotids is an area where there is a clear advantage to 3.0T over 1.5T. The increase in field strength yields improved image quality that translates to improved reproducibility and better identification of plaque components. SAR may be a problem in some black-blood vascular imaging sequences due to the large number of 180° pulses used.

## Conclusion

Performing CMR studies at 3.0T compared to 1.5T causes a number of important physical changes. Foremost among these changes is an increase in bulk magnetization which results in increased SNR. Additionally, changes in the resonant frequency produce increased RF power deposition and an increase in inhomogeneities of the RF excitation field. Changes in the relaxation times require changes in timing parameters that depend on T1. The increase in the main field strength causes an increase in the effect of magnetic susceptibility artifacts.

Several CMR applications have been shown to improve at 3.0T. The majority of improvements are due to the increase in SNR, but some improvements are due to the change in tissue T1 values. Challenges remain to the widespread use of 3.0T for all cardiac applications. The major limitations are the increased artifacts seen in SSFP imaging and the increased SAR of common sequences. It is important to note that 3.0T protocols have not had time to be optimized in the way 1.5T protocols have been over the past 10 years. It is clear that simply using 1.5T protocol at 3.0T may not yield the most optimal results. New developments in parallel imaging, new sequence developments, and further protocol optimization will be required to realize the complete benefits of CMR at 3.0T.

## Competing interests

John Oshinski has received research funding from Philips Medical Systems and Siemens Medical Solutions.

## Authors' contributions

JNO, JGD, PS, AG, RIP participated in drafting of the manuscript.
